# How hepatitis C patients manage the treatment process of pegylated interferon and ribavirin therapy: a qualitative study

**DOI:** 10.1186/s12913-016-1503-6

**Published:** 2016-07-11

**Authors:** Shu-Mei Tsai, Jung-Ta Kao, Yun-Fang Tsai

**Affiliations:** Graduate Institute of Clinical Medical Sciences, College of Medicine, Chang Gung University, 259, Wen-Hwa 1st Road, Tao-Yuan, 333 Taiwan; School of Medicine, College of Medicine, China Medical University; Division of Hepato-Gastroenterology, Department of Internal Medicine, China Medical University Hospital, 2, Yuh-Der Road, Taichung, 404 Taiwan; School of Nursing, College of Medicine, Chang Gung University; Department of Nursing, Chang Gung University of Science and Technology; Department of Psychiatry, Chang Gung Memorial Hospital at Keelung, 259, Wen-Hwa 1st Road, Tao-Yuan, 333 Taiwan

**Keywords:** Antiviral treatment, Employing strategies, Hepatitis C experience

## Abstract

**Background:**

Hepatitis C virus (HCV) infection is a global public health issue. Adequate treatment for hepatitis C patients is important, but anticipated side effects make patients fearful of receiving treatment. Little is known about the experiences of hepatitis C patients who have completed treatment with pegylated interferon and ribavirin. The purpose of this study was to explore the experiences of hepatitis C patients who had undergone therapy with pegylated interferon and ribavirin and gain an understanding of what factors contributed to completion of treatment.

**Method:**

This was a qualitative study with 21 adult hepatitis C patients purposively sampled from outpatient liver clinics of a medical university hospital in Taichung City, Taiwan. Participants had completed 6–12 months of therapy with pegylated interferon and ribavirin. Data were collected through individual, face-to-face, in-depth interviews conducted in the participants’ homes from June–October 2013. Data were analysed using conventional content analysis.

**Results:**

Data analysis revealed three themes that described the strategies employed to alleviate and ease symptoms and manage the processes involved: restructuring their lifestyle, adopting a positive attitude, and seeking support.

**Conclusion:**

Hepatitis C patients face many challenges during treatment with pegylated interferon and ribavirin. These findings provide knowledge that can be used in designing effective programs to help other Hepatitis C patients manage the side effects of pegylated interferon and ribavirin therapy, complete treatment and improve quality of life.

## Background

Hepatitis C virus (HCV) infection is a serious health problem; 130–150 million people globally have chronic hepatitis C infection [1]. The prevalence of HCV infection in most countries is 1–2 %, however in Taiwan it has been reported to be as high as 4.4 % [2]. One reason for the higher level of HCV infection in Taiwan may be insufficient medical resources: failure to use disposable medical devices, blood transfusions (blood donors were not screened for antibody to HCV in Taiwan before 1992) and the injection of drugs using shared needles [3]. There is no available vaccine preventing HCV infection. Among HCV-infected patients, only 20 % retain normal liver function and most of them will face an increased risk of hepatocellular carcinoma (HCC) (odds ratio of 35) in the future [4].

Antiviral therapy can increase the cure of HCV infection [1, 5]. Consequently, adequate treatment for HCV patients is critical. Before 2011, pegylated interferon and ribavirin (PEG/RBV) was the standard for HCV treatment. The treatment duration is 24 or 48 weeks depending upon such factors as viral genotype, baseline serum HCV RNA level, and fibrosis score on liver biopsy [6]. During the past five years, owing to the rapid development of new anti-HCV drugs, direct-acting antivirals (DAAs) have been approved for HCV treatment [7, 8].

A positive sustained virologic response (SVR), which is an undetectable HCV viral load after treatment for 6 months, defines successful therapy for HCV infection [9]. The SVR rate with PEG/RBV is approximately 70–80 % for HCV genotypes 2/3 and 45–60 % for genotypes 1/4 [5]. More recently, the second generation of DAAs has enabled interferon-free therapy, which has increased the SVR rate to more than 90 % for genotype 1 [8, 10, 11]. However, concerns remain about virologic relapse after completion of therapy and the extremely high cost of these drugs in many countries [8, 12–14]. Therefore, PEG/RBV remains the standard treatment for HCV in numerous countries, including Taiwan [10].

There are adverse side effects of PEG/RBV therapy, which impact patients on several levels. The physical side effects (nausea, fatigue, anemia, neuropsychiatric) are unpleasant, sometimes intolerable, and are often the major reason for discontinuation of hepatitis C treatment [15, 16]. The adverse effects have a broad impact on patients: approximately 40 % of patients are required to reduce the dosage, and 10–15 % choose to cease therapy [11]; in addition patients experience a decreased health-related quality of life (HRQOL) [17, 18]. Therefore, side effects from the therapy of PEG/RBV require close monitoring in order to reduce the possibility of discontinuing treatment.

Most studies have focused on the physiological and psychological side effects of PEG/RBV therapy and the influence on a patient’s HRQOL, but little is known about how patients who complete treatment manage these side effects. Understanding how HCV patients successfully complete the therapy is central to gaining information on effective strategies for coping with the discomforts of treatment. Many studies have explored the patient's experience of treatment for hepatitis C in Western countries [19–22], yet few reports exist for Eastern countries. The purpose of this study was to explore the experiences of HCV patients in Taiwan who had completed PEG/RBV therapy in order to obtain an understanding from a different cultural perspective of how the treatment process was managed. Our findings can be used to design patient education programs to teach effective strategies for managing the obstacles of HCV treatment and support for other HCV patients intending to undergo such therapy in Taiwan and other countries.

## Method

### Design

We used a qualitative design with individual, face-to-face, in-depth interviews to explore the experience of treatment for hepatitis C with PEG/RBV from the patient’s viewpoint. HCV patients who had completed combination therapy were invited to share their experiences of the treatment process.

### Participants

Study participants were purposefully sampled from outpatient liver clinics at a university hospital in Taichung City, Taiwan. Participants were included by these criteria: ≥ 20 years, chronic HCV patients who had completed 6–12 months (24–48 weeks) of PEG/RBV therapy, and able to communicate in Chinese or Mandarin. Patients were excluded if they had communication difficulties or severe cognitive deficits. Eligible patients were approached at the liver clinic by their hepatologist who explained the study and introduced interested patients to the first author. The first author explained the study purpose, the amount of time required, the necessity to audio record the interviews, the strategies used to protect confidentiality and privacy, and entitlement to withdraw from the study at any time and to refuse to answer questions for any reason. Participants signed a consent form prior to the interview. The study was reviewed and approved by the Research Ethics Committee of China Medical University & Hospital (CMUH102-REC3-058).

### Data collection

An interview guide (Table 1) was designed especially for this study, which consisted of six open-ended questions to assist with the interview process and allow the participants to share their experiences in more detail. The questions were developed in consultation with a hepatologist, and experts in medical care. Two HCV patients who had completed treatment pre-tested the interview guide, which was revised as a result of their opinions. The pilot interviews were not integrated into the analysis. Demographic data were also collected at the interview.

**Table 1:** Interview guide

1. How did you know that you were infected with hepatitis C? 2. What interests or worries did you have about starting treatment? 3. What kinds of symptoms from combination therapy for hepatitis C did you have? 4. Please describe your experience. How has combination therapy affected your life? 5. How did you deal with the symptoms of hepatitis C with combination therapy? 6. Please describe your experience. What was the most important factor that supported you in facing the treatment process? Why?	

Data were collected from June–October 2013 in semi-structured interviews conducted by the first author, trained in interviewing and with 12 years of clinical experience in medical and surgical nursing. Interviews were scheduled at a time convenient for the participant. Individual, face-to-face, in-depth interviews were conducted in the participant’s home and lasted between 45–120 minutes. Participants were recruited for the study until no new codes emerged from the interviews (data saturation) [23].

### Data analysis

The audio recordings were transcribed verbatim within 48 hours of the interviews, and transcripts were analyzed by inductive content analysis. We employed conventional content analysis for our data, which is generally used with a study design whose aim is to describe a phenomenon. This type of design is usually appropriate when existing theory or research literature on a phenomenon is limited. Analysis starts with reading all data repeatedly to achieve immersion, and coding categories are derived directly from the text data [24]. Data were collected simultaneously with data analysis and continued until no new information emerged. We employed a qualitative and inductive coding process [25].

The narrative transcripts were analysed by multiple readings; two authors independently read the transcripts and developed an initial list of open codes and memos to derive categories from raw data. All three authors participated in peer discussions, constantly checked the coding consistency and revised the coding rules continually until sufficient coding consistency was achieved [26]. Finally, highly related codes were integrated into categories and the same core meanings of the categories were defined as themes. After data analysis was completed, results were translated into English.

We enhanced rigor and trustworthiness of the study by employing the four criteria of Lincoln and Guba [27]. Credibility was established in several ways: peer debriefing through discussions of general methodology, transcripts and analysis with two experts in qualitative research with 13–20 years of clinical experience; pre-testing of the interview guide by two HCV patients who had completed combination therapy treatment; providing a private space for the interviews (scheduled at a time convenient for the participants); and prolonged engagement was established through the physician, who had cared for the participants for three to four years in the clinic. To establish dependability, an audit trail of all procedural steps and methodological decisions was maintained. Confirmability of the transcripts was assured by using multiple investigators. Two of the authors reviewed the coding data and theme labels to ensure that results were data-driven, and then all three authors discussed any differences until consistency was achieved. Transferability was met through purposive sampling.

## Results

### Participant characteristics

Findings are based on interviews with 21 participants (13 females, 8 males) who had completed PEG/RBV therapy. The mean duration of HCV treatment was 8.4 months (SD = 3.1). Participants’ ages ranged from 35–83 years (mean = 60.86; SD = 11.47); the majority had a junior high school education or higher (48 %), were married (59 %) and 90 % lived with their families (Table 2).

**Table 2:** Participants’ characteristics (*N* = 21)^*^

Participant	Education	Living with family	Marital status	Employed	Treatment duration, months
1	Junior high school	Yes	Divorced	No	12
2	University	Yes	Married	No	6
3	Junior high school	Yes	Widowed	No	12
4	Junior high school	Yes	Married	No	12
5	Primary school	Yes	Married	No	12
6	Senior high school	Yes	Married	No	6
7	Primary school	Yes	Widowed	No	12
8	Primary school	Yes	Married	Yes	6
9	University	Yes	Married	Yes	12
10	Uneducated	Yes	Widowed	No	6
11	Primary school	Yes	Widowed	No	6
12	University	Solitary	Unmarried	Yes	6
13	University	Yes	Married	Yes	6
14	Primary school	Solitary	Widowed	Yes	6
15	Primary school	Yes	Married	No	12
16	Primary school	Yes	Married	No	6
17	Uneducated	Yes	Married	No	6
18	Primary school	Yes	Married	No	6
19	Primary school	Yes	Widowed	No	12
20	University	Yes	Married	Yes	6
21	Junior high school	Yes	Married	Yes	12

^*^Participants =13 females, 8 males; ages ranged from 35–83 years (mean = 60.86; SD = 11.47)

### Employing proactive strategies

Patients receiving the therapy with PEG/RBV for HCV reported experiencing numerous uncomfortable symptoms during treatment. In order to manage these difficulties they employed several strategies. At the core of all of these strategies was a proactive approach in order to take charge of the side effects of treatment. Analysis of the data from the transcripts identified three themes that further described these strategies: ‘restructuring of lifestyle’, ‘adopting a positive attitude’ and ‘seeking support’.

### Restructuring of lifestyle

To cope with and adapt to the difficulties of HCV treatment participants altered their lifestyle: they changed their diet, added physical exercise to their daily routine, modified daily habits they believed detrimental to their health and added complementary and alternative medicine (CAM). Participants believed a healthy lifestyle could protect liver function and prevent further liver damage.

Food plays an important role in Chinese culture and some patients were willing to change established patterns such as eating habits and alcohol consumption in order to reduce the physical discomfort of treatment and improve symptoms related to HCV. One participant stated:

*I used to drink alcohol, but now I rarely drink…I changed my eating habits. I eat eggs and beef for my breakfast and lunch, then at dinner I try not to eat meat to give my stomach a rest… I drink a cup of Kucha oil in the early morning before I have eaten anything… (P2)* One participant discontinued observing a dietary restriction to improve nutrition: *“Because of the traditional Chinese custom, I cannot eat beef. However, in order to improve the symptoms of anemia, I have to eat beef…”* (P7)

Restructuring physical activities or learning new skills helped to manage the discomfort. One patient stated, *“Almost three times a week, I would go to the gym to do exercise and use the sauna and spa…”* (P8) One participant learned how to self-inject, “*Because I needed the injection with interferon every week, I learned how to inject it myself…”* (P6)

Eastern medicine is readily available in Taiwan and CAM played an important part of therapy. Participants employed massage therapy, acupuncture, and Chinese herbal medicine to relieve their discomfort and improve their strength. One patient reported, “*When I feel uncomfortable, I go to see the Chinese Medicine doctor, and I have acupuncture…And for my health, I use Chinese herbal medicine every day.”* (P5) Another used massage therapy, saying, “*I went for a Chinese massage once a week… Massage made me relax and feel comfortable.”* (P3) Herbal tea helped another: “*I drank Ganoderma Lucidum Tea every day; I heard Ganoderma Lucidum has an effect on the immune system and liver protection*.” (P17)

### Adopting a positive attitude

A positive attitude helped participants manage the discomforts of treatment. Rather than allow the difficulties of treatment to weigh on their emotions, they turned their situation into one that was constructive. Some compared their own experiences of treatment with those suffering from other diseases, which provided them with confidence to confront and accept treatment. A patient previously treated for breast cancer believed HCV treatment was much easier, stating, “*I had surgery and chemotherapy for breast cancer, so I‘m not afraid of death…Compared with my cancer experience, you will see which is better.”* (P11) Another participant focused on diseases with worse outcomes, which bolstered confidence saying, “*Compared to patients with serious diseases, I thought ‘I’m so lucky’…Then I wasn’t afraid to face difficulties from the treatment process.”* (P21)

A positive outlook on life allowed some participants to confront obstacles optimistically; they believed that positive thinking was a positive force. The knowledge that HCV was treatable was an effective incentive. One participant expressed this by stating, *“I felt strange about hepatitis C. I thought there was no way to treat it. I felt hopeless…Then, I learned that hepatitis C can be treated—this good information made me hopeful about my life…”* (P2) Another said, “*I thought, ‘I am still young’, I felt confident about the future…so I received the treatment.”* (P13)

### Seeking support

Support refers to an individual’s subjective perception of having assistance available or being cared for. During treatment participants sought out different forms of support: informational support, emotional support and financial support. Participants explained that acquiring more information about HCV allowed them to better understand the process of treatment:

*I got an information pamphlet about hepatitis C from the hospital; it provided me with some information…I gained more knowledge about hepatitis C, I was not afraid of the disease and its treatment…Sometimes I looked up information about hepatitis C on the Internet.* (P20)

Emotional support of family, doctors, nurses, and friends encouraged participants to face the challenges of treatment. Physical symptoms were eased by support from a nurse and doctor: “*Every time I went to the clinic, the nurse smiled at me and encouraged me…I felt pleased…The doctor told me ‘Go! Go!’ I thought, ‘I will fight off hepatitis C’ … I forgot the uncomfortable symptoms of treatment.”* (P6) A nursing friend was a source of information as well as motivation: “*My friend is a nurse, so she has been a great help to me…If I have any questions, I asked my friend or the doctor…She always encouraged me.”* (P18) A doctor and family members provided comfort to another participant:

*When I went to the clinic, the doctor was concerned for my condition, and I felt very comfortable …My son worked in Tainan, but he called me every day…My three children were very concerned about me. It made me feel warm…* (P3)

One of the significant side effects of treatment for HCV is the nausea and fatigue, which can interfere with patients’ daily self-care abilities and responsibilities. Family members who could care for the patient and assume their responsibilities were critical to continuing treatment. A wife’s spouse supported her when she was unable to continue with daily activities: “*I felt weak; I had no strength to hold a glass… Fortunately, my husband took care of me…My husband helped me do housework such as hanging the clothes, cooking, cleaning the house…”* (P4)

For those who were employed (33 %), disruption of work required financial support. Some participants suffered job loss due to frequently missing work to receive treatment. Treatment costs and the reduction of income were heavy financial burdens that also required family support. One patient best described this by stating:

*I was a mason. When I begin to receive the treatment; I must request sick leave every week… Finally, I submitted my resignation. Then, my finances became worse. I must rely on my daughter to support the daily living expenses. (P8)*

Participants expressed gratitude for the financial support provided by health insurance. National health insurance was implemented in Taiwan in 1995, which alleviated much of the financial burden of healthcare. One patient described the importance of this support: “*The cost of treating hepatitis C is paid for by the National Health Insurance, which lets us save the cost of more than $3000 dollars and reduces our economic burden…*” (P10)

## Discussion

This study highlights the experiences of 21 HCV patients who completed treatment with PEG/RBV for hepatitis C in Taiwan. Participants reported being worried about beginning therapy; they thought it would be impossible to endure and were extremely afraid. However, adopting proactive strategies to confront the adverse effects of HCV therapy helped them endure the 24–48 weeks of treatment. Several approaches were utilized: restructuring of lifestyle, adopting a positive attitude, and seeking support. To our knowledge, this is the first time an application of these strategies has been described for HCV patients in an Eastern country.

The standard treatment strategy was based on the therapy of PEG/RBV for HCV patients, which was the only therapy available until 2011. The recent appearance of DAAs has changed HCV therapy. The new treatments offer all-oral regimens, short duration of therapy (from 6–12 weeks to 24–48 weeks), few side effects, and high cure rates (>90 %) [28]. However, use of these new treatments is restricted in many areas of Asia, because of the high cost [29]. Although the first generation of DAAs was approved for clinical use in 2011, data regarding the long-term virologic response relative to genotype and disease status is unclear [29]. In addition, treatment outcomes with PEG/RBV therapy are better in Asian than non-Asian populations due to factors such as IL28B genotype, low body weight, and HCV genotype misclassification [30]. Therefore, PEG/RBV is still the main standard therapy for HCV patients in Asian countries such as Taiwan.

An added difficulty for our participants was the significant financial burden resulting from the high cost of treatment. The median cost for HCV treatment with triple therapy (first generation DAA/ PEG/RBV) has been reported to be nearly $84,000 (US dollars) per patient in New York [31]. In Taiwan, national health insurance eased some of this burden resulting from treatment with PEG/RBV. However, because patients are cared for by family members, there is a loss of income not only as a result of job loss for patients, but also from a reduction in work hours for family members, and these adverse events added to financial worries.

Taking a proactive approach to restructuring their lifestyle helped participants manage the adverse physical effects of treatment, while at the same time improving their overall health. The most common changes were altering dietary habits and increasing activity levels. The importance of a healthy diet was emphasized, which included vitamins and other supplements. They also stressed the importance of a diet high in vegetables and fruits, and avoided drinking alcohol. Consistent with our study, Stoller et al. [32] showed that HCV patients in the Midwestern U.S. modified diet and activity level, and made use of CAM to manage the physical and psychosocial consequences of HCV infection. Exercise was also included in restructuring the participants’ lives. Controlled studies evaluating the effectiveness of exercise on reducing discomfort and improving HRQOL for HCV patients undergoing PEG/RBV therapy are suggested.

The use of CAM is growing globally. Nearly half of the participants (47.6 %) reported using CAM as a medical self-care strategy [33]. Because of cultural differences and the availability of CAM in Taiwan, participants in this study may have been more likely to use Eastern medicine to minimize the discomfort of therapy. This was especially true for the female participants, which may be due findings that women are 4 times more susceptible to decreases in hemoglobin during treatment with PEG/RBV [34]. The participants’ experiences with Chinese massage, acupuncture, and Chinese herbal medicine resulted in reduced side effects and thus improved their HRQOL. Chronic disease patients have used CAM to improve symptoms and HRQOL during and after treatment in other studies [32, 35]. Additional research evaluating the efficacy of CAM therapies for hepatitis C patients should be considered.

A positive attitude empowered some participants with emotional strength and the confidence that they could endure the arduous course of treatment. In our study, older participants drew on experience from past illnesses, the awareness that some illnesses produce even greater discomfort and the understanding that hepatitis C was treatable. Conversely, younger people applied a positive approach to manage their stress.

For many participants, support was an important component for managing treatment. Knowledge about hepatitis C was helpful; participants who were more highly educated searched the Internet frequently for information about HCV. Some of these participants suggested that healthcare professionals could assist HCV patients by designing a dedicated hepatitis C Website. Fraenkel et al. [35] reported that patients valued support groups to cope with the adverse events of treatment. Formal support resources for patients and their families are lacking in Taiwan. Availability of support groups and development of a social-network Website containing hepatitis C information would allow patients and their families in the same situation to share their experiences.

One significant difference between hepatitis C patients in our study and those in Western cultures is most participants (90 %) lived with their families; families were the most common sources of support. A multitude of physical and emotional side effects occurred during therapy; participants’ roles shifted from independence to dependence and normal daily routines became burdensome, which also affected their job. Family members were an important source of physical, psychosocial, and financial support, which is consistent with findings reported by Fraenkel et al. [35]. Family members are often the primary caregivers of hepatitis C patients in Taiwan, therefore it is important that appropriate and adequate information about hepatitis C be made available to the family.

This study provides new insight into treatment experiences of patients with CHC in Taiwan who completed PEG/RBV therapy. Knowledge regarding these participants’ experiences could help other HCV-infected patients take a proactive approach to develop effective strategies for dealing with the many side effects, aid in preparedness for therapy and improve chances for completing treatment.

### Limitations

Despite its contributions, there were two limitations to this study. First, we recruited only participants who had completed PEG/RBV therapy (to increase homogeneity), which may have contributed to their unique perspectives. Future studies might consider including patients who had discontinued HCV treatment, since such patients can augment these findings by understanding what prevented patients from completing treatment. Second, this study only interviewed patients; data could be enriched and strengthened with multiple data resources. Because patients’ family members and healthcare providers played an important supportive role during treatment, they could offer additional insight regarding the process of treatment. Therefore, future studies might consider enrolling these support members to enrich the data.

## Conclusion

Treatment of chronic hepatitis C is a challenging process; it is known to produce significant physiological and psychological side effects, in addition to financial strains. The participants in our study shared the strategies they employed to manage these adversities during treatment. Knowledge gained from their experiences can offer healthcare providers insights concerning effective management skills that can help other HCV patients complete PEG/RBV therapy. A patient-centered care approach is important for providing support to this unique patient population. Therefore, we recommend that healthcare providers build a patient-centered care model through multidisciplinary cooperation (hepatologist, psychiatrist, nurse, social worker, dietician, etc.) to provide care and support for HCV patients during the treatment period.

## Abbreviations

CAM, alternative medicine; CHC, chronic hepatitis C; DAAs, direct-acting antivirals; HCC, hepatocellular carcinoma; HCV, Hepatitis C virus infection; HRQOL, health-related quality of life; PEG/RBV, pegylated interferon and ribavirin; SVR, virologic response rate
